# “Empathy for children is often missing”: a mixed methods analysis of a German forum on COVID-19 pandemic measures

**DOI:** 10.1186/s12889-024-20296-0

**Published:** 2024-10-12

**Authors:** Văn Kính Nguyễn, Astrid Berner-Rodoreda, Nina Baum, Till Bärnighausen

**Affiliations:** 1 Heidelberg Institute for Global Health, Heidelberg University, Heidelberg, Germany; 2 Harvard Center for Population and Development Studies, Cambridge, MA USA; 3 Africa Health Research Institute, KwaZulu-Natal, Durban, South Africa; 4 Visiting Researcher, Imperial College London, London, UK

**Keywords:** Pandemic preparedness, COVID-19, Vaccination, Children, Families, Embeddings, Clustering, Qualitative

## Abstract

**Background:**

The pandemic and its preventive measures disrupted daily routines and posed unforeseen obstacles for families. Users of public online forums chronicled these challenges by freely expressing their sentiments in unrestricted text-length formats. We explored a German COVID-19 forum to understand family perspectives and experiences of pandemic measures, particularly in terms of testing and vaccinating children. Our findings aim to inform future epidemic health policies.

**Methods:**

We retrieved all 11,207 entries from a COVID-19 forum during its lifespan (June 2020 - December 2021), posted during the height of the pandemic. We classified the entries into topic clusters including general pandemic situation, testing, or vaccination using state-of-the-art text embeddings and clustering algorithms. The clusters were selected based on the research’s aims and analysed qualitatively using a health policy triangle framework.

**Results:**

Users generally appreciated pandemic public health safety measures for everyone’s protection, yet voiced concerns about inconsistent policies and disproportional disadvantages for children compared to other societal groups, like the elderly. Non-compliers were overwhelmingly regarded with skepticism and critiqued. Users found COVID-19 (exit) strategies and information about the benefits of vaccination unclear. This created hurdles for parents and caregivers in navigating their children’s school and social life. Users endorsed vaccinating children mainly for “normalising” children’s lives rather than for their physical health benefits. Some users suggested prioritising teachers and early childhood educators on the vaccination eligibility list to speed up a return to “normality”.

**Conclusions:**

During pandemics, governments should prioritize addressing the societal and mental health needs of children by implementing participatory and family-oriented public health measures for schools and kindergartens. Clear communication coupled with consistent design and implementation of safety measures and regulations, would be crucial for building trust in the general population and for ensuring compliance regarding testing and vaccination. Communicating the benefits and risks of vaccinating children is of paramount importance  for informed decision-making among parents. In future epidemics, computer-aided analysis of large online qualitative data would offer valuable insights into public sentiments and concerns, enabling proactive and adaptive epidemic responses.

**Supplementary Information:**

The online version contains supplementary material available at 10.1186/s12889-024-20296-0.

## Background

 The COVID-19 pandemic impacted people worldwide [1–3]. Parents, caregivers, and children were particularly affected [4], as the pandemic disrupted daily routines and led to a home-centred situation [5]. With schools and daycare centres closed or operating at limited capacity, parents had to juggle their jobs and their children’s education and care [6, 7]. While studies underscored quality family time during lockdowns, increased stress and exhaustion [8] as well as negative impact on the mental health of caregivers has also been reported [9, 10]. Social distancing and closure of recreational facilities limited children’s socialisation [11] and physical activities [12]; at the same time, increased sedentary behaviour affected children’s physical [13, 14] and mental health [15]. Given the lingering threat of the next epidemic [16], understanding the experiences of families and children during the COVID-19 pandemic is crucial for pandemic preparedness.

Two critical measures for an effective epidemic prevention and response are testing to detect new cases early and increasing vaccination coverage to achieve the epidemic control threshold [17]. For COVID-19, the estimated herd immunity threshold ranged 65–89%, depending on the variants and vaccination strategy [18]. However, COVID-19 vaccination coverage has been low in children and young people compared to other age groups. In the UK, it ranged from 14.5 to 52.5% for the age groups 5–11 and 12–17 year-olds [19] respectively; in Germany, the percentages were 22.5% and 74.6% [20] respectively. While there are many studies on adult determinants of vaccine delay or refusal, such as perceived vaccine safety, vulnerability, and trust in the healthcare system [21], the few factors which have been identified in relation to unvaccinated children were unvaccinated family members and number of children in the household [19]. Conducting explorative qualitative studies will facilitate further quantitative assessments of variables that may be associated with unvaccinated children. Both approaches will contribute to a better understanding of this  phenomenon. 

To this end, we focused on data from a Brigitte family internet forum. Brigitte is a German magazine that has been in circulation since 1886. It covers diverse topics from fashion to health and politics.  95% of the readership is female; 62% are between 30 and 59 years old, and with an average net income of 3.784€/month [22]. Its digital version reached 7.66 million unique users as of 2022 [23]; 72% of users are women and over 50% are below the age of 49 [23]. As an online forum, internet access is a precondition. In Germany, internet access coverage is 95–98%; 85% of the age groups 25–64 year-olds have access to the internet [24]. Brigitte forum users show a wide age range and geographic distribution. In June 2020, early in the epidemic, a dedicated Brigitte forum was created entitled “Coronavirus - child, family, kita, school, in times of corona [sic] pandemic” to discuss family, children and schooling in times of COVID-19, thus appealing to parental users and  providing a rich source of information for exploring issues related to children during the epidemic.

This paper aims to draw lessons learnt from this internet discussion forum to gain deeper insights into how families were affected by COVID-19 and how health messages, particularly regarding testing and vaccination were received and acted upon, with a view to inform future epidemic health policies. This study also provides a showcase for the analysis of large amounts of text through combining quantitative textual analysis for content identification and filtering with human qualitative analyses and interpretations.

## Materials & methods

**Fig. 1:**
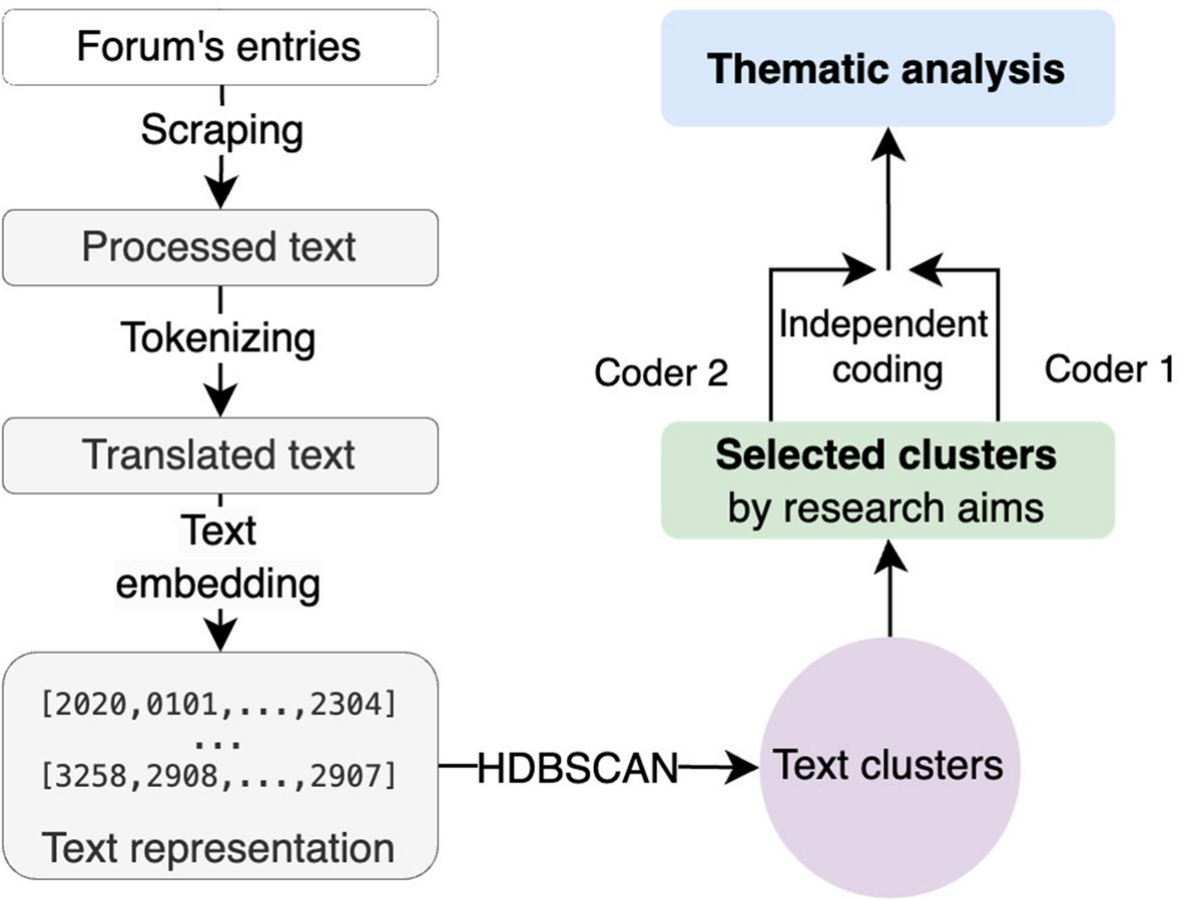
Schematic of the analysis flow. The public forum data were retrieved and cleaned before transforming into high-dimensional numerical representation for the data clustering methods, upon which targeted topics are extracted and analysed thematically

We combined strengths from novel machine learning textual analysis techniques and qualitative methods to analyse the large volume of text more efficiently, as shown in Fig. 1 and described in detailed below.

### Data processing

We programmed in R and Python to get the full extent of the forum discussion through web-scraping, i.e., automatically accessing all the discussion pages, parsing the context using the HTML parser BeautifulSoup [25] and saving the information. This included the time, username, main message, and (possibly multiple) quoted texts for each of the posted messages. We recorded emoticons by extracting the title of the icons as they are not in a compatible encoding format (UTF-8) and converted them to the equivalent format to factor in the emoticons’ sentiment; the mapping table is available on Github repository (kklot/netnography). The messages were systematically split into individual sentences (tokenised) before sending them through DeepL.com’s API for translation from German to English (setting the post request’s option to split_sentences = 0 to prevent unwanted post-processing by DeepL). In order to have the highest sentence segmentation accuracy for the German language, we used the spacyr::spacy_tokenize function with the trained models (de_dep_news_trf).

### Cluster analyses

Based on the translated text, embedding vectors, a numeric representation of the extracted contents, were created for each message using the state-of-the-art pre-trained model text-embedding-ada-002 from OpenAI [26]. The distance between embedding vectors measures the relatedness of text strings and was used for text clustering. Dimensions of the data were analysed using the unsupervised t-distributed Stochastic Neighbor Embedding (t-SNE) [27]. Based on that, we used the hierarchical density-based spatial clustering of applications with noise HDBSCAN [28] to group messages into clusters based on the density and proximity to each other calculated from the text embedding above; we calibrated the HDBSCAN’s parameters to look for a smaller number of clusters with dense information.

### Thematic analysis

Our underlying theoretical framework was the health policy triangle developed by Walt and Gilson who posit that content, context and process as well as actors are central “to plan for more effective implementation” [29]. Based on the identification of thematic clusters above, we focused on entries regarding the pandemic situation at the beginning and end of the forum (1349 and 643 entries, respectively), attitudes towards testing (276 entries), and vaccinations, with a cluster focusing on teachers (155 entries) and other clusters focusing on children in early and late 2021 (166 and 193 entries, respectively). Entries could vary from single sentences to a number of paragraphs. ABR and NB entered the data into NVivo 12 Pro. They initially coded 50–70 entries independently: ABR from the start and NB from the end of the dataset. A joint codebook was established by comparing the codes and further adjusted as they coded more forum extracts.

For further datasets, 20 entries were initially coded separately and compared to determine if new codes were necessary. The two coders then divided each dataset for coding, with 100 entries assigned alternately to each coder. ABR and NB compared between 60 entries and the entire dataset, depending on its size, and jointly agreed on the final codes for each entry. The codes described the situation or testing practices and also referred to sentiments such as attitudes towards school openings and closures, attitudes towards vaccinating children and vaccinations in general, and discussions on quarantine issues, infectiousness, and incoherent political decisions. An example of coding is provided in the supplementary material Table 1.

As is standard in fora, users select a pseudonym as username which also obscures the gender of the user; to avoid unexpected identification, we altered the username if it had a real-sounding name or nickname. This alteration is annotated with a superscripted star. We also removed numeric parts to the name. With the closure of the forum in December 2021, users could change their status to “inactive user”, which is abbreviated here as “IU”. Direct quotes from the forum were translated into English.

## Results

During the forum’s duration (June 2020-December 2021), user activity peaked at the end of the first wave of the COVID-19 pandemic. As the pandemic progressed, the discussion waned but was reactivated with each increase in COVID-19 cases (Supplemental Figure S1). The forum primarily featured personal experiences and discussions about the pandemic, including public health measures in various German states and other countries. On average, five users participated in the forum discussion daily. Overall, users posted 11,207 messages, totalling six million characters (including emoticons).

### Message clustering

**Fig. 2:**
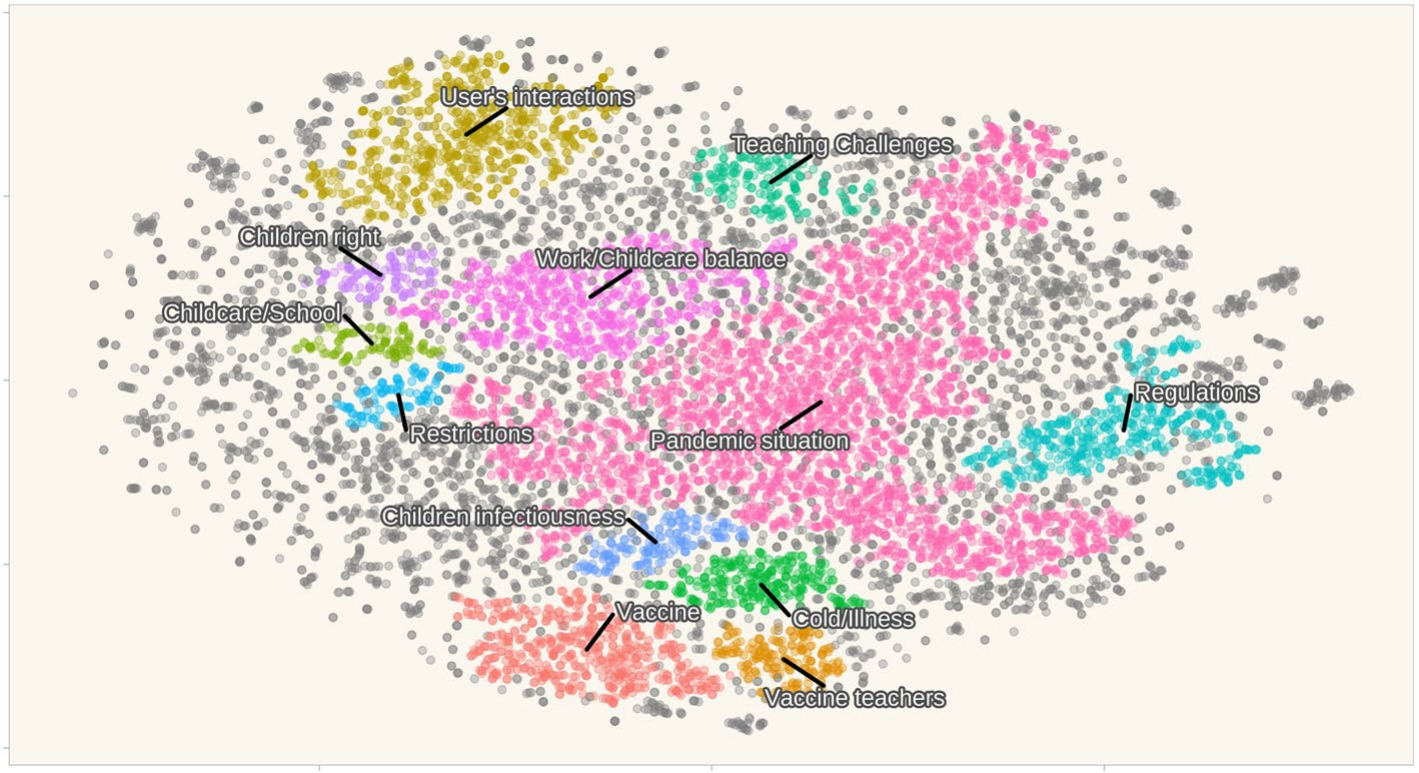
Overview of major discussion topic clusters of the whole dataset. Each point represents a posted message (11207 in total). Points with the same colour belong to the same cluster classified by the proximity of the text embedding vector via HDBSCAN; grey points indicate outliers that were not assigned to the identified clusters. The axes are coordinates calculated using the t-SNE dimension reduction based on the embeddings calculated from text messages. The cluster labels were named for illustration purposes by summarising the messages within each cluster to find the most prominent topic of discussion

Fig. 2 provides an overview of the forum topics by presenting eleven main thematic clusters identified by clustering techniques. We summarized all messages within each cluster to generate the most prominent topic of the cluster. As a focused forum, most cluster topics interrelated, showing few outliers (entries that do not form a group or belong to an identified group). This implies that the discussions are focused, connected, and rich in information about the topics. Most messages were about the pandemic situation, followed by discussions on balancing childcare, work-life, regulations, and vaccinations. The 'interactions' cluster denotes users agreeing, disagreeing, or otherwise engaging with each other’s messages.

**Fig. 3:**
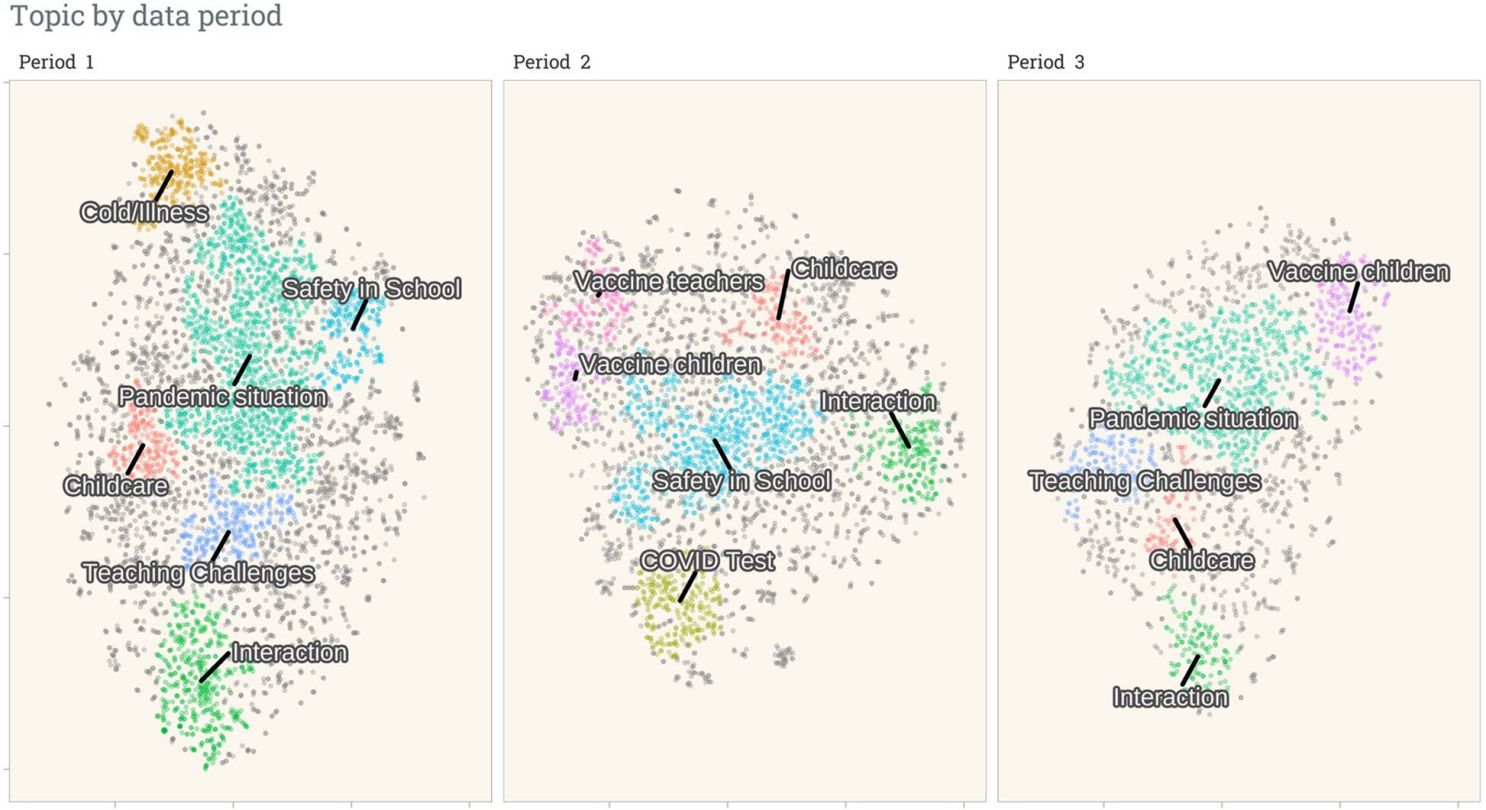
Clusters of major discussion topics are separated by three distinct periods: from June 2020 to 28 Jan 2021 (period 1), to 15 July 2021 (period 2), and to 12 December 2021 (period 3). Each point represents a posted message (11207 in total). Points with the same colour belong to the same cluster; grey indicates outliers not assigned to the identified clusters. The axes are coordinates calculated using the t-SNE dimension reduction based on the embeddings calculated from the messages. The cluster labels were named for illustration purposes by summarising the messages within each cluster to find the most prominent topic of discussion

In order to identify entries and clusters that are relevant to testing and vaccinating children, we split the data into three discussion periods which closely followed the pandemic waves. These periods roughly correspond to (1) testing services being slowly expanded but no vaccination services for COVID-19, (2) testing services in operation and the early rollingout of the COVID-19 vaccines, and (3) vaccines for children being approved (Supplemental Figure S1). We applied the clustering techniques on each of the split datasets and extracted the relevant clusters to the research question, including pandemic situation, vaccines, and COVID-19 testing (Fig. 3).

Figure 3 shows that the clusters are still in close proximity but more distinct clusters can be seen, such as COVID test and vaccination, denoting a concentrated discussion of those topics. A thematic shift can also be observed over the three half-yearly periods: the pandemic situation was a topic for the first and third periods, and discussions on vaccination and testing only emerged in 2021, corresponding with the roll-out of vaccinations in Germany on December 2020 [30, 31]. Based on these topics of interest, we extracted the messages of the clusters relevant to the research question and thematically analysed the content. In the following section, we will present the themes we identified through a qualitative analysis. 

### Thematic discussions

Generally, most users were favourably inclined towards following public health regulations. Yet, they critiqued inconsistencies in implementing the regulations, not just in terms of variability in different German states but also in having stricter measures applied to schools than elsewhere in society with children bearing the brunt of the pandemic. From the entries provided, it seemed that the majority were mothers. Some wrote from the perspective of grandparents, others self-identified as teachers. While most were based in Germany, some users also wrote about the situation in Switzerland, Austria, and Spain.

We will present findings according to the identified datasets of the pandemic situation (2020; late 2021), testing (early 2021) and vaccination (2021).

#### The pandemic situation: “my children… are not the drivers of the pandemic” (inactive user)

In 2020, pandemic forum discussions revolved around children’s ability to pursue their hobbies, attitudes towards school-related public health measures, and suggesting solutions for re-establishing some normality. The following issues were paramount in terms of number of entries (in brackets): incoherent political decisions (444), wish for reliable schooling (227), understanding the COVID-19 pandemic and infectiousness (193), description of life under the pandemic (175), quarantine regulations and practice (115) and the burden of parents (103 entries). The most active users in terms of the number of posts for the early pandemic dataset were Wolkenschaf, Stern* and Margarine* and the aggregated number of inactive users (IU) who had deactivated their accounts after the forum was closed. In 2021, Stern* became more active than Wolkenschaf, with Margarine* playing a less prominent role.

##### Children bear the brunt

Users felt that authorities applied more severe rules to schools than to the rest of society. Bars, driving and music schools could open, and people were allowed to celebrate weddings or parties, go on holidays and demonstrate while schools remained closed. Also, for older people, life normalised more quickly:

And I personally found it actually absurd that my parents (over 80) were leading an almost completely unrestricted life sometime in early summer (except for having to wear masks, but that applies everywhere) with vernissages, restaurant visits and choir singing, while my children were still sitting at home. (IU)

Children were seen as lacking representation or advocacy, unlike older people, who were considered an important voting demographic. Blame for the situation was directed at various levels, including politicians, education and state ministries, school principals, scientists, and health authorities. The prevailing sentiment was that clear strategies for sustainably re-establishing school life were lacking. Additionally, schools, teachers, and some families were ill-equipped for remote teaching. Users highlighted educational deficits and the social and psychological impact on children. Instead of focusing on providing reliable schooling, teachers had to prioritize emergency care for a few children, leaving less time for the rest of the class. Although alternate schooling was not the preferred model, users saw it as a better option than no schooling or continuing with burdensome home-schooling. “It’s clear that over a longer period of time, one can hardly manage to work from home and assist one or more children with their digital lessons at the same time” (Elixier*).

##### Inconsistent and non-enforced rules

Users wanted authorities to implement regulations in all contexts, whether at schools, during teacher-parents’ evenings, or across the general population. “Mask wearing is compulsory, but it is not enforced” (Mandarine*). At the same time, users noted regulatory inconsistencies, such as, a decrease in COVID-19 cases not leading to a reopening of schools or children being allowed to mingle freely in the morning at school but required to maintain distance in the afternoon. “And the probability (of an infection) is higher after school than at school?” (Stern^*^).

##### Infectiousness of children

Differing user views on the infectiousness of younger children compared to older children sparked debate. “Primary school children are certainly not drivers (of the pandemic), whereas 16-18-year-olds or older should be seen like adults” (IU). Users cited studies showing differing degrees of infectiousness of children. “According to this, the infection rates are ‘above the average for school children of all ages’, even in primary school” (Alaska). The matter remained inconclusive in the forum.

##### Coping as a family

Users described family situations and exchanged information on regional incidence, lockdowns, quarantine measures, extended forced school holidays, and the impact of public health measures. Quarantine rules were felt to be particularly non-sensical.

Our child was a KP1 (contact in the childcare centre with a positively tested early childhood teacher). The sibling was supposed to go to school as normal, and we were supposed to go to work (which works really well with the kindergarten child looking after him/herself at home). (Cayenne*)

The focus of the discussion was on children’s well-being and learning. Users were concerned about the psycho-social impact of their children not spending time with their friends. “…I am not even so much concerned about what they learn at school, rather I would want my child to experience everyday life with ‘his peers’ again” (IU). Last-minute decisions on school closures impacted on work and family organisation, and made it nearly impossible for parents to balance work and assisting children with home-schooling or managing lunch preparations. Yet, some also valued the positive side of spending meals together at home, going for walks, and children learning more independently.

##### Logistical challenges and hope for the future

In 2021, safety remained a dominant concern. However, the availability of vaccinations and self-tests raised users’ hopes for re-establishing “normality” in pursuing activities even if life was more cumbersome.

I can understand that one finds the 3G rule from the age of six annoying if one has children under 12. But don’t you feel more comfortable in a swimming pool/cinema when you know that everyone in here over the age of six is vaccinated, has recovered from COVID-19 or is tested, and therefore probably not a COVID-19 transmitter? (IU)

The possibility of daily self-tests had changed the quarantine rules in some locations: “Classes are no longer sent into quarantine in the event of a positive case; this [quarantine] is replaced by 5 days of daily testing” (IU). Being quarantined after returning from holidays was, however, viewed as putting children who were ineligible for vaccination at a disadvantage:

I am very much with you on the issue of quarantine: it is very unfortunate that children now have to spend five days in quarantine if they have been on vacation in a high-risk area with their vaccinated parents. I think testing is ok, but then for everyone, please. (Stern^*^)

Mothers complained about having to organise the testing equipment, document the testing, and ensure the child takes or brings back all the signed documents: “I find the logistics huge right now, and I’m generally well organised” (Stern^*^).

The Omicron strain of the virus was perceived as more dangerous for children: “But I am no longer convinced that children are barely at risk” (Margarine*), yet the greater challenge for many remained the schooling situation: “The possibility of an infection doesn’t give me sleepless nights, but the thought of school and childcare closures does” (Wolkenschaf).

While many users supported the reopening of schools, some felt that this required additional safety measures. New approaches like teaching outside the school building or using university students as reinforcement and crediting the teaching to their teacher’s training should be tested. They expressed hope that vaccines might normalize life. “My children are currently being vaccinated, so in a fortnight we could not care less whether there are COVID-19 cases in the class or grade” (IU).

#### COVID-19 testing: “If life is halfway normal with a lot of testing - then bring it on” (IU)

In addition to the testing dataset of early 2021, testing was also mentioned in the vaccine and pandemic situation datasets. Users were generally positively disposed towards testing despite the additional burden of conducting the tests. The most active users in the testing dataset were Stern*, Malaita, Wolkenschaf, and Blue, as well as those who had later changed their status to “inactive”. Discussion centred around child-friendliness of various test types and test procedures, whether tests are a helpful measure for children, the pros and cons of different test locations and whether testing should be compulsory. There was general mistrust in some parents testing their children at home. Users felt that stricter measures were necessary for parents and children who did not comply with testing requirements.

##### Attitudes towards testing children

Users generally had a favourable view of testing children, seeing it as a means to detect asymptomatic cases, reduce viral transmission, facilitate contact with grandparents, and keep schools open. They felt that children could cope with the tests. “I don’t think testing is bad for my first grader or my 5-year-old. It would be worse if everything closed again” (Wolkenschaf).

Some users favoured harder measures if children could re-experience “normal” school life. “I think it’s great that there is now compulsory testing here at secondary schools. Yesterday my big one had his first, all negative, and everyone had a lot of fun together during wild PE lessons afterwards“ (Mandarine*). The exclusion of non-compliers was also seen positively. “But anyone who refuses to be tested cannot take part in certain activities. Children must then remain in home schooling*“* (IU). Yet, some expressed mistrust in the behaviour of other parents. “I can only reiterate that after - unfortunately not only isolated cases of - parental feedback on compulsory testing, I really have strong doubts that tests would be handled honestly and responsibly at home*“* (Stern*). Others empathised with reluctant parents as a positive test result could affect their ability to work.

With primary school children, the procedure is much more difficult, because the parents would all have to be on stand-by in case of a positive result in the classroom, so I understand that parents are not happy about it if they don’t work from home and don’t have a workplace that they can simply leave. (Franzbrötchen*)

Stigma and trauma were additional considerations. “And then I asked myself, especially with the first grader, what testing triggers in him. Far from being traumatised, but it’s not to be taken lightly” (IU).

##### Place of testing

The attitude towards testing was closely connected to a discussion on testing location: at school, at home, at a testing centre, or at the paediatrician’s office. In terms of convenience, users preferred testing at home or school; the latter was considered more reliable than at home but raised stigma concerns. While many testing centres did not test children, testing at a paediatrician was felt to be a burden in terms of time and organisation.

What is currently stressing me a bit is that although I have lots of tests at home for my children, they are of no use to me because I am not allowed to test my 3-year-old with a runny nose at home and the kindergarten believing the result. Instead, I have to take her to the paediatrician. Last week it took over two hours, including the half-hour wait. Very pleasant experience with a 10-week-old baby in tow. (Gender*)

##### Test friendliness and reliability

Many users preferred lollipop and spitting tests to deep throat and nasal tests. “I really don’t know if I would put my son through two “deep” tests every week like his first PCR test. At the announcement of a further test, he totally shut down and started crying” (IU). Yet, opinions varied. “They might even find it quite funny to poke their nose with a stick. (I fear that’s the case.)” (IU). The discussion also focused on the reliability of the tests and whether there was any interaction with food or drinks. “We also test nasally, and I know of several false positive cases where it was suspected that it was due to juice or sweets consumed shortly beforehand” (Stern*). A further source of unreliability was the way children may conduct them.

I find the combination of suboptimal sample collection (no deep swab) and suboptimal test (antigen instead of PCR) in asymptomatic children not so great. Either the more thorough sampling or the more sensitive test… But of course it is still better than no test at all (if it is done regularly). (Minstrel)

##### Open questions and incoherent testing policies

Some issues remained vague for users, such as at what age and where to test young children, leading to “‘dealing’ with addresses for testing children under the age of six 🙄 in kindergarten” (Cayenne^*^). Clear procedures were also missing after a positive self-test.

What about the time between the rapid test and the PCR result? Do you also have to go into quarantine? Personally, that would be a genuine reason for me to refuse. I don’t want to be locked up time and again for no reason. (IU)

Similar to the datasets on the pandemic situation, political decisions were felt to be incoherent in that only school children were continually tested, not adults.

First of all, I would be in favour of all employees being OBLIGED to be tested regularly instead of employers just “providing” the tests benevolently. What’s the point of that? Testing is compulsory in schools, at least in my state. (Malaita)

The number of tests required per week were felt not to correspond with the state of the pandemic. Users also wanted antigen tests conducted in schools to be recognised by other institutions. Instead, they had to additionally test younger children for a haircut, or leisure activities like visiting the zoo or cinema. Some users felt overwhelmed by the testing requirements and believed vaccinating the entire family would be a better solution.

As the big one won’t be 15 until August, and with a bit of luck we parents will be vaccinated at the end of next week (with Johnson&Johnson, so we would be through after one vaccination), we will actually be having seven test-free weeks in the summer if we manage to get the vaccination (IU).

#### COVID-19 vaccinations: “If I were to have the children vaccinated, I would do it for practical reasons rather than out of a fear of COVID-19” (Wolkenschaf)

Vaccinations against COVID-19 were offered to the general population in Germany at the start of 2021 [32]. While the pandemic datasets had relatively few entries on vaccinations, we identified three vaccination-related datasets – two with a focus on children in early and late 2021 and one with a focus on teachers in the early part of 2021. The most active users in the vaccination datasets were Wolkenschaf, Ministrel, Stern*, and Margarine* and the ones that later deactivated their accounts, i.e., the “inactive users”. Prominent themes were attitudes towards vaccinating children (211 entries), personal experiences with vaccinations (142 entries), and general attitudes and feelings towards vaccinations (92).

In the first half of 2021, users revealed a positive attitude towards vaccinations. Still, they did not see the need for a compulsory COVID-19 vaccination nor a real need to vaccinate children against COVID-19 if all adults were vaccinated. Users shared information about how and where to access vaccinations. Additionally, they believed that teachers and parents should be given higher priority in the vaccination schedule. The entries showed little empathy for non-vaxxers and expressed the hope of vaccinations being offered to everyone. No clear position emerged on whether people should have to reveal their vaccination status or how to prioritise vaccines globally. Many users were willing to vaccinate children above 12 years of age but expressed uncertainty about whether children below 12 years should be vaccinated. Users relied on and respected the recommendations by Germany’s Standing Committee on Vaccination (STIKO). Reasons for vaccinating children were not only seen in protecting vulnerable groups such as the elderly and having a milder course of infection but also in re-establishing “normality” for children.

##### Nuanced understanding of vaccinations

Users discussed the benefits of vaccinations and herd immunity, also with regard to other infectious diseases such as measles.

There are diseases that are SIGNIFICANTLY more contagious than COVID-19, such as measles, and yet herd immunity is possible. … COVID-19 is less highly infectious, so the vaccination rate for herd immunity must also be less high. (IU)

Breakthrough infections were regarded as inevitable, not a counterargument to vaccinations.

There is far too little talk about which groups in particular have breakthrough infections. I am also convinced that this is the case, and I find it logical. When anti-vaxxers speak out, they often say that there are many breakthrough infections, as if it’s nonsense to be vaccinated. (Malaita)

##### Uncertainties about vaccinating children

Whether or not to vaccinate children was a dominant discussion theme. Perceived advantages were the benefits of protecting children with pre-existing conditions and preventing transmission to others, especially given the potential for asymptomatic infections. “It’s deceptive that the little ones get infected but have no symptoms - and merrily pass on the virus. Vaccinated children can protect many people” (IU). Potential long-term effects were considered a drawback for vaccinating children. Most users expressed confidence in STIKO recommendations and appreciated the independent evaluation of studies. The opinions of paediatricians also played an important role. Despite a general inclination towards vaccination among many users, when the possibility of vaccinating children increased in late 2021, so did the uncertainty about children’s risk/benefit ratios.

I am actually almost always team ‘vaccination’ but in view of the short period since authorisation, the understandable STIKO decision, not to make a recommendation so far as well as the fact that the second Biontec [Pfizer] vaccination has affected me a little more and longer and I am not the only one, I would find it difficult to have a child vaccinated who, at age 12, probably has half my body weight at best. On the other hand, I still think vaccination is the only way forward, and my syringe-hating daughter declares that she would like to be vaccinated (but of course. she can’t really grasp all the arguments at the moment). (Stern*)

Users perceived little to no benefit of the vaccination for children’s own health. While several parental users were ambivalent, others felt that vaccination brought a lot of practical benefits in forgoing quarantine and mask-wearing. This tendency was even more pronounced in late 2021. Those who had vaccinated their children hoped to re-establish their social life: “My big one has had his first vaccination. He will be able to take part in everything again even before Christmas.” (Mandarine^*^). Some users became impatient: “I can’t wait for the vaccination for children. And we will vaccinate as soon as EMA has authorised it and not wait until the STIKO has finished its dilly-dallying.” (IU). Parents were also motivated to vaccinate their children due to concerns about Long COVID.

##### Children are not epidemic stopgaps for vaccine-avert adults

Users believed that the responsibility for getting vaccinated lay first and foremost with adults - “children are barely at risk and it is therefore not imperative to vaccinate them; especially not for the protection of adults who are unwilling to be vaccinated” (IU). Adult non-vaxxers were perceived extremely critically: “The unvaccinated just make me puke” (Rock^*^). While parents who hesitated to vaccinate their younger children experienced empathy, unwilling adults were seen as not pulling their weight in containing the epidemic through vaccinations. Yet others felt that epidemics could only be contained if *everyone* got vaccinated: “On the other hand, everyone is part of the herd to be immunised, so all children must be vaccinated just like all adults” (IU).

##### Viewing teachers with empathy and skepticism

The exchange revealed that only Baden-Württemberg, the southwestern state, prioritised teachers for vaccinations. Many users felt that teachers and early childhood educators should be among the first to get vaccinated because their work with children was important; vaccinating them would also help protect children who were not yet eligible for vaccination. Users, including teachers and early childhood educators, voiced the expectation that professionals in the education sector should get vaccinated and that refusing to do so was anti-social:

But what about the early childhood teachers who don’t want to get vaccinated? Will they be ‘allowed’ to go into quarantine for 14 days if a case occurs and I have to cover for my colleague? That can’t be right either. (IU)

A few users expressed empathy for individuals who personally chose not to get vaccinated:

Based on reports that women who want to conceive should rather not be vaccinated, there were also comments from this age group in my childcare centre that they were thinking about whether they should do it. I totally understand that.” (IU).

Overall, users expected personnel in the health and educational fields to consider the public good rather than their individual preferences. For those unwilling, users felt that authorities should assign different tasks. “It’s OK, if early childhood teachers don’t want to be vaccinated, of course. But by implication, it would make sense to then take them out of the groups ” (IU). Alternatively, they should not receive any pay for their quarantine time. “Unpaid leave of absence would really be an adequate quarantine means for unvaccinated early childhood teachers. I know, it will not happen that way” (Wolkenschaf).

The ensuing discussion centred around the timing of the vaccination for teachers to minimise absenteeism at school or early childhood centres. Users’ opinions varied, with some viewing educational personnel as lazy for taking precautionary sick days immediately after their scheduled vaccination dates. “Oh my, instead of rejoicing and hoping for the best, one calls in sick as a precaution. Main thing is to be fit again in the holidays” (Hoop^*^). Others, including teachers, highlighted that they acted responsibly:

I am a primary school teacher and will be vaccinated next Saturday, the first day of the holidays. Like many of my colleagues. We were NOT able to choose the date, but we are all happy that we can recover from possible side effects during the holidays. We take our responsibility seriously and know how valuable every teaching day is at the moment. Apart from that, there are a significant number of my colleagues who are getting vaccinated because they don’t want to be absent from school in the future. (IU)

Other users sympathized with the difficulty of securing vaccination appointments. They felt that coordinating these appointments at the school level and using a staggered approach to minimise school closures was too complex and demanding. “And headmaster Müller is then to organise that Mrs Maier and Mr Schmidt should please be vaccinated four days later so that no lessons are cancelled??? How is that supposed to work? (IU). The fact that teachers received the vaccination was more important to them: “One does not have to make such a fuss about a few lessons being cancelled because of a vaccination appointment.” (Malaita).

In late 2021, users discussed particular vaccines and expressed preferences towards Biontech/Pfizer or J&J and empathy with teachers for receiving Astra Zeneca vaccines: “Teachers are usually vaccinated with Astra Zeneca, and I have often heard that side effects in the form of fever and fatigue frequently occur afterwards” (IU).

##### Outlook

In early 2021, users hoped for vaccinations restoring normality. “Vaccination helps (age-wise) top down. And as soon as enough people have been vaccinated, you should open up from the bottom for all those who are at very low risk anyway” (Minstrel). In late 2021, one user expressed the hope for a child-friendly vaccination in form of a nasal spray. Yet, overall, users perceived the virus as becoming endemic with little chance of achieving herd immunity due to anti-vaxxers and their children. Users feared a persistence of public health measures: “Should we continue like this for years? You can’t ruin a generation of children because of this” (Wolkenschaf).

**Fig. 4:**
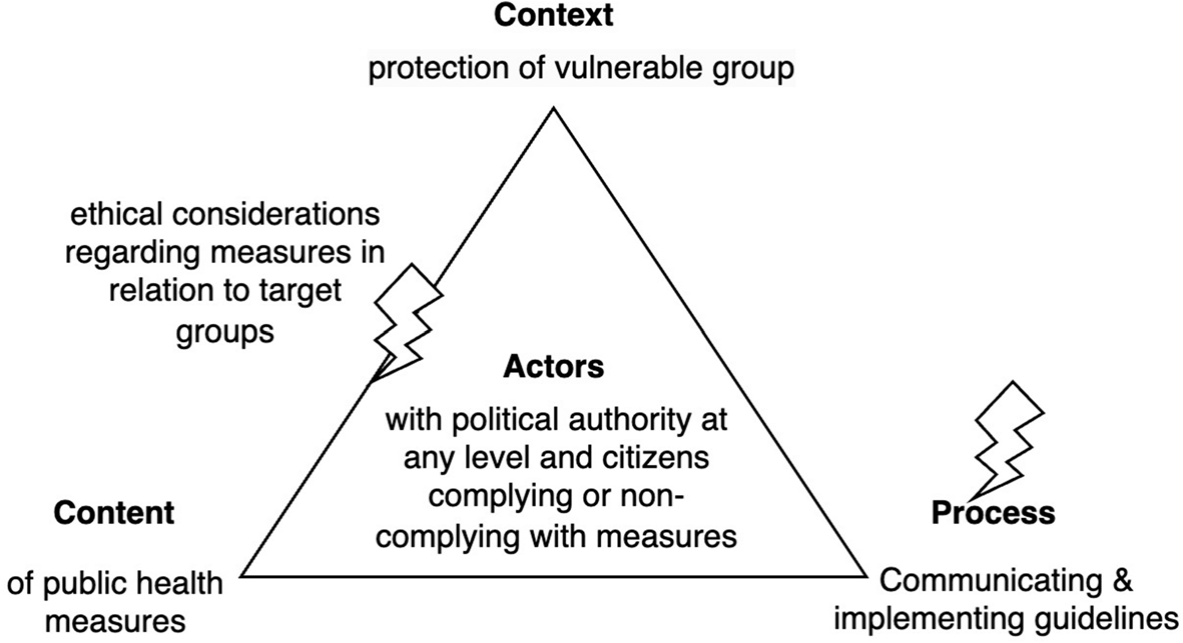
Covid-19 public health triangle as perceived by forum users

We will analyse these findings in relation to the health policy triangle [29] which we have adapted as follows, see Fig. 4.

## Discussion

Our analysis of a German COVID-19 online forum on childcare and family life (Brigitte Forum) is based on a combination of clustering techniques and qualitative methods and identified prominent topics in users’ exchanges about public health measures and their impact on children. The forum discussion spanned over a year and a half during the pandemic which was characterised in Germany by long school closures and uncertain schooling with gradually more self-testing measures and availability of vaccinations, first for adults, then for children. The results showed users' openness to testing and vaccinations to overcome the epidemic and normalize life, as well as the expectation towards others to behave responsibly. Users also showed respect for government bodies for vaccination recommendations, but struggled with inconsistent policies that affected their lives and the lives of their children; they also grappled with the decision whether or not to vaccinate younger children. The wish to re-establish normality for children was paramount.

We have adapted the health policy triangle (see Fig. 4) for our analysis and interpretation, as the forum provided a parental perspective of COVID-19 policies.

### Who was seen as a political actor and how were they perceived?

Political actors, as seen by forum users, ranged from politicians from chancellor level to scientists providing advice, STIKO, local authorities, school principals and teachers. Forum users appreciated political decision-makers who proposed measures that seemed to help overcome the epidemic quickly but critiqued those that seemed to make their lives and that of their children more complicated through haphazard school openings, time-intensive and uncoordinated testing, unclear quarantine procedures and inconsistent implementation of measures introduced. The view of the actors was thus intricately bound to the content and the process of implementing public measures – with some users showing understanding for complicated political processes and the limits of decision-making at local level and others judging ineffective political decision-making more harshly. Bodies that provided advice, such as STIKO, were greatly appreciated, mostly because of their independent scientific examination of evidence. In the view of users, that gave their recommendations more credence. Another important group for users were local paediatricians who they knew and trusted. German online surveys in 2020 showed trust to be the most influential factor for accepting COVID-19 policies [33], and a US study found the paediatrician “ the most trusted source of information about COVID-19 vaccines” for children [34].

Users also perceived their own role as actors in the implementation of public health measures, as compliance or non-compliance would affect the duration of the epidemic. Notably, users underscored the importance of adhering to public health measures, expressed positive attitudes towards testing and vaccination, and criticized individuals who refused to test or get vaccinated. These sentiments stand out in the context of global misinformation spread by anti-vaxxers [35] and protests organised by outspoken groups [36]. In Germany, protests were linked to the so-called “Querdenker” groups, which criticize political decisions and oppose restrictions on their rights and “freedoms”. The majority of these groups are anti-vaxxers [37]. While a local survey at a Southern German demonstration did not link the Querdenker to right-wing groups (ibid), others emphasised their closeness with right extremists [38, 39] and the tendency to “normalise” extreme right-wing positions [40], particularly in areas where the far right has a stronghold [41]. Querdenker may have also instigated resistance to COVID-19 measures in other countries [42] and recruited children into online groups [43]. The danger of hijacking political agendas was clearly seen by forum users who did not share any Querdenker sentiments. The forum seemed to attract parents, grandparents, and teachers who belong to the silent majority rather than fringe groups. Interestingly, Jäckle and colleagues found in their German survey studies that “the positive effect of political trust is significantly stronger for liberal respondents and weaker for more authoritarian persons” [33].

### Context and content – what should be considered in the next epidemic?

With an aged and ageing population at risk of severe COVID-19 outcomes [44], Germany prioritized the protection of the elderly and those with pre-existing medical conditions through periods of home confinement and quarantine measures as well as population public health measures ranging from physical distancing, mask-wearing, testing, lockdowns, school closures, travel restrictions to vaccinations [45]. Measures affected older people in various ways ranging from isolation and loneliness to coping relatively well [46], showing that older people are not a homogenous group. While forum users perceived the need to protect the elderly and vulnerable, the longer the measures lasted and the more freedoms the older generation regained (church, choir, restaurant visits), users felt that the context had shifted to favour the older population to the detriment of their children whose schools remained closed. This was described by a user as a “technocratic gerontocracy” and echoes literature critiquing the disproportional burden of public health measures on children [15, 47, 48]. While the context of the next epidemic and the population groups that may be most affected is still unclear, it will serve well to also consider those that bear the brunt without being prime beneficiaries of the measures.

With periodic new guidelines issued by Germany’s public health institute RKI, and users researching and citing studies and STIKO recommendation updates [49] as well as recommendations from other countries, they showed familiarity with concepts such as R-rates, mutations, the benefit of testing, tracing and social distancing, and the effect of vaccinations on the epidemic and vaccine breakthroughs. This seems to compare favourably to a survey in Germany’s first COVID-19 wave where only 50% had good COVID-19 related health literacy for understanding COVID-19 information, even though 90% felt well-informed [50]. Brigitte (forum) users may be generally slightly better educated than the average German, and this may have facilitated a deeper understanding of public health measures.

#### What can be improved in the testing strategy?

As Germany had produced the first PCR test for detecting SARS-CoV-2 [45], testing played an important role in the German epidemic control and was generally appreciated by forum users as a public health measure. Forum users argued for testing children at home to reduce potential stigma and for testing children at school with potentially more reliable test results and more transparent school quarantine policies, reflecting the findings of other studies [51]. Users were aware, however, that tests conducted at schools required additional personnel resources and that at-home or self-tests may not be conducted or documented accurately. Developing a general confirmation and validation of the test result would ease the administrative impact on the public. Users also wished for school test results to be acknowledged by other institutions. Testing strategies should include wider test result validity so that tests, irrespective of the place of testing, would be recognised elsewhere for a limited period, particularly as some testing centres were not testing children. In addition, users felt that testing should be conducted in a child-friendly manner. As the reliability of self-tests was questioned, a list of recommended test kits in terms of specificity and sensitivity and information about which tests to use for what purpose (antigen vs. PCR) should be communicated to families (parents and children) and children’s supporting networks including kindergartens, schools and leisure institutions to successfully implement the testing strategy.

#### Prioritising population groups for vaccination

In any epidemic with limited access to vaccines, prioritising access is unavoidable. Some users criticised that teachers in most German states did not receive a high enough priority position, given their essential role in society [32]. Open schools contributed up to 20% of population transmission even under hygiene and testing measures, particularly during the spread of the omicron variant in early 2022 and could be as low as 2% at other times [52]. Further simulation and modelling studies should demonstrate the contributions of different vaccination priority strategies [53] to pandemic control, and results should inform the prioritisation order across states.

#### Ethical considerations at the juncture of context and content

Re-establishing normality through vaccinations was an option for adults - notwithstanding access to vaccination appointment challenges. It was initially not an option for children as vaccines had not been approved for younger children [54]. While prioritizing vulnerable groups such as the elderly and those with pre-existing illnesses was necessary to reduce incidence and mortality [31], the socio-psychological effects of the COVID-19 measures were keenly felt by children. They experienced high rates of depressive and anxiety symptoms and sleep disturbances [55] as well as increasing household poverty rates [56].

The dominant discussion on the infectiousness of younger children and whether or not infectiousness was linked to particular viral strains, reflected evidence available at the time [32, 57, 58] and led users to weigh up the benefits and risks of vaccinating their children. The fact that parental forum users talked about their own COVID-19 vaccination but hesitated to vaccinate their younger children as they were unsure of the safety and side effects of the vaccine is also echoed in parental surveys in Israel [59, 60]. Nonetheless, users’ decision to vaccinate their children was driven by the desire for fewer restrictions, the reopening of schools, and restoring their children’s school interactions rather than solely aiming to prevent a severe infection in their children. As such, parents vaccinated their children less for the child’s physical health benefits but rather for social benefits in re-establishing some kind of “normality”. Users also felt that children were being enlisted to contribute to herd immunity because adults were unwilling to be vaccinated, effectively “using” children for the benefit of adults. The unwillingness and individual freedom of some adult individuals not to be vaccinated was thus perceived as jeopardising the health of the masses [61]. The forum discussion highlighted ethical concerns regarding fairness and justice in vaccinating a group primarily for the health benefit of other groups rather than their own. In research, the justice principles uphold that one group should not bear the costs for the sake of another [62]. Does this principle apply to vaccinations? While some argue that it is justifiable to have children vaccinated for the common good and herd immunity [63], others contend that in the case of COVID-19, it was ethically unjustifiable to put pressure on children to be vaccinated [64]. Critical ethical considerations to be addressed by ethics councils in future pandemics should examine the infectiousness of particular sub-groups such as children and weigh up own vaccination versus population benefits.

### The process

The initiation and implementation of policies and guidelines was for many users the area that needed most improvement. They pointed out inconsistencies across states and cities – what was allowed in one place, was prohibited in another. While users appreciated different epidemic situations in different locations, it was difficult to convey to a general population that schools are open in state x and closed in state y; also, the unequal treatment of population sub-groups was critiqued (old vs. young; restaurants open, schools closed); not following through with the implementation of guidelines, e.g., demanding the wearing of masks and then tolerating that people do not wear them was felt to be counterproductive. Complex quarantine regulations that were impractical for families and seemed to keep changing were felt to discourage people from testing, and social distancing measures differing at school from meeting the same people in the afternoon seemed non-sensical. Non-enforcement of public health measures, inconsistencies and the proportionality of measures were also critiqued in a Canadian qualitative study [65] and in a German qualitative interview study with parents and children [66]. In order to get the buy-in from the general population, in this case parents, school children and teachers, it is important to include representatives in the drawing up of guidelines and in deciding how these guidelines are going to be implemented at the various political levels – from federal to state to communal and school levels. This may help in ironing out inconsistencies in the guidelines and improving implementation.

### Strengths and limitations

We analysed public health measures from the perspectives of the governed not of the governing. While the Brigitte forum has a wide reach, the users are not a representative sample of the parent population as users self-selected to participate in the forum. However, the forum provided a unique, rich andunprompted account of users' experiences from various regions in Germany and beyond. Compared to social networks, traditional forum entries have more anonymity as users do not need to provide personal information. They are also less likely to come from a fake account as the forum represents a special interest group. Without knowing for sure the gender of the user, we assume that we mostly read mothers’ entries; experiences of fathers may thus be underreported in this analysis. The cluster visualization shows that the entries were highly focused and connected on the forum topics.

We used state-of-the-art textual analysis and clustering approaches to digest the large volume of text collected. The methods accelerate the targeted analysis of important topics and the discovery of relevant topics in the entries. In contrast to common text analysis approaches, the text classification was conducted based on the full paragraph instead of keywords or sentence tokenisation, thus considering the context of the whole entry and allowing a better grouping of the message with similar topics. As subsequently confirmed via manual coding by authors ABR and NB, the entries within each cluster largely refer to the cluster theme yet also cover other cluster themes. This challenge can be expected as the topics within the pandemic context are somewhat interrelated.

While the entries within each cluster were ordered chronologically, they did not constitute immediate reactions to preceding entries, which made it difficult to appreciate some sentiments. As manual thematic coding and analysis are time-intensive and impractical for large datasets, AI-led coding, which can cover data quickly but may not consider the same nuances, should be further investigated. The retrospective analysis of the entire forum data (which had been closed at the time of analysis) allowed all the aspects of the discussions to be captured and classified. We would, however, recommend following and analysing online discussions as they emerge. This may provide further insights into individual user’s experiences and observations on relationships forming between users. We would therefore recommend additional studies to be conducted in life fora.

### Conclusion and outlook

We presented the assessment of COVID-19 health measures from the perspectives of a segment of the German population, i.e., mostly parental users of an internet forum. While the entries showed that families struggled with public health measures, users' overarching motivation was to overcome the pandemic and keep people safe by endorsing measures that pave the way for normality, especially for their children. Using the health policy triangle, we highlighted issues that would require targeted and inclusive planning for future epidemics. Specifically, context must be considered to avoid disadvantaging those who comply with measures that protect others. Additionally, involving target groups in defining content and processes may lead to measures that all actors can endorse.

Exit strategies for epidemics should prioritise the mental and social well-being of children alongside crucial indicators like incidence and mortality rates. This would involve developing targeted strategies for a return to a “normal life” for children and their support networks. Research should investigate impacts of different strategies for epidemic controls in schools to contribute to more evidence-based policies and strategies [67]. Additionally, exit strategies should aim to restore “normalcy” across different population subgroups, particularly those who are less affected by epidemic transmission.

## Supplementary Information

Supplementary Material 1.

## Data Availability

The datasets collected and analysed during the current study are available in Github’s kklot/netnography repository.
