# Supplementary material for: “Empathy for children is often missing”: a mixed methods analysis of a German forum on COVID-19 pandemic measures
Source: BMC Public Health. 2024 Oct 12;24:2795. doi: 10.1186/s12889-024-20296-0 (PMC11470561; doi:10.1186/s12889-024-20296-0)
Supplement: Supplementary file 1 — Supplementary Material 1. [file 12889_2024_20296_MOESM1_ESM.pdf]

Supplemental Information

“Empathy for children is often missing”: A mixed methods analysis of a German forum on COVID-19 pandemic measures

Văn Kính Nguyễn*[1,4,+], Astrid Berner-Rodoreda*[1], Nina Baum[1], Till Bärnighausen[1,2,3]

* equally contributed

[1] Heidelberg Institute for Global Health, Heidelberg University, Germany

[2] Harvard Center for Population and Development Studies, Cambridge, MA, USA

[3] Africa Health Research Institute, KwaZulu-Natal, South Africa

[4] Visiting Researcher, Imperial College London, United Kingdom

[+] Corresponding to kinh.nguyen@uni-heidelberg.de

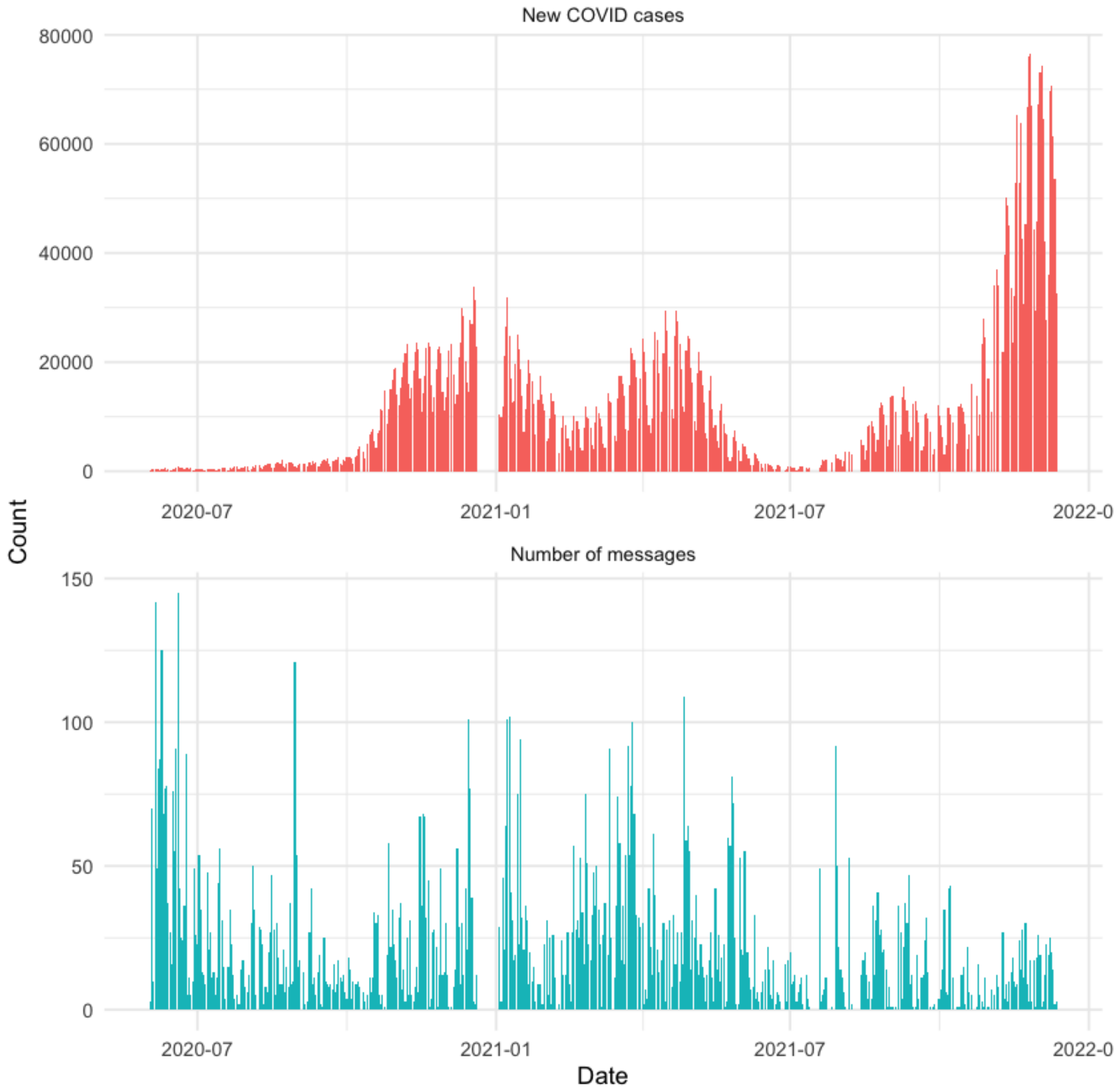

Figure S1. Count of messages per day and COVID-19 incidences.

Supplementary Table S1 : Example of coding for themes across datasets

Main theme	Sub-theme	Codes	Dataset	Topics Discussed	Quotes (Translation)
**Vaccination**	**Uncertainties about vaccinating children**<br>(summary: general positive attitude towards vaccinations; desirable to have herd immunity but doubtful if it can be achieved or if children are necessary because of adult antivaxxers; Uncertainty and hesitancy about benefits of vaccinating smaller children (<12 years);<br>main reason for vaccinating children: re-establishing a “normal” life for them)	Attitude towards vaccinations in general (positive and negative feelings about any vaccination for any disease, effectiveness, impact of vaccinations on diseases)	2nd wave vaccine and teachers	Positive attitude dominant;<br>Free decision by teachers/early childhood educators, but unvaccinated should bear consequences.	Vaccinations mean safe protection; herd immunity is important and it is not okay that institutions (schools, kindergartens) have to be closed time again for quarantine because of these people.
			2nd wave vaccine and children	Positive attitude dominant; protecting self and others; more respect for COVID-19 than adverse vaccination effects. Some speak about life’s risks	Vaccinated, I protect myself and also children- that’s the way I see it.
			3rd wave vaccine and children	Positive attitude dominant; vaccination protects against outbreaks and viral mutations; discussion about booster; no understanding of antivaxxers;<br>some speak about pers. experience of vaccination breakthroughs and reactions to vaccinations.	I would get vaccinated again, even with the risk that there might be complications.
		Attitude towards vaccinating children (positive and negative feelings about vaccinating children)	2nd wave vaccine and teachers	Uncertainty about pros and cons of vaccinating children;<br>doctors/pediatricians have differing opinions	And when the vaccination is approved for children? Are you going to let your children be vaccinated?
			2nd wave vaccine and children	Discussion when there might be vaccine for children; positive attitude towards vaccinating older children; weighing up pros and cons for younger children	I still find that it is not so clear to weigh up the risks of the vaccination for children.
			3rd wave vaccine and children	Waiting for STIKO recommendation for smaller children; users feeling queasy but would get them vaccinated to re-establish normality. For older children, vaccination okay.	The question of whether they (children) have any benefit. If I were to have the children vaccinated, I would do it for practical reasons rather than out of a fear of COVID-19.
